# Human Lectins and Their Roles in Viral Infections

**DOI:** 10.3390/molecules20022229

**Published:** 2015-01-29

**Authors:** Christopher P. Mason, Alexander W. Tarr

**Affiliations:** School of Life Sciences and Biomedical Research Unit in Gastroenterology, Faculty of Medicine and Health Sciences, University of Nottingham, Nottingham NG7 2UH, UK; E-Mail: nixcm@exmail.nottingham.ac.uk

**Keywords:** innate immunity, lectin, HIV, hepatitis viruses, therapeutics, mannose binding lectin, ficolin, DC-SIGN

## Abstract

Innate recognition of virus proteins is an important component of the immune response to viral pathogens. A component of this immune recognition is the family of lectins; pattern recognition receptors (PRRs) that recognise viral pathogen-associated molecular patterns (PAMPs) including viral glycoproteins. In this review we discuss the contribution of soluble and membrane-associated PRRs to immunity against virus pathogens, and the potential role of these molecules in facilitating virus replication. These processes are illustrated with examples of viruses including human immunodeficiency virus (HIV), hepatitis C virus (HCV) and Ebola virus (EBOV). We focus on the structure, function and genetics of the well-characterised C-type lectin mannose-binding lectin, the ficolins, and the membrane-bound CD209 proteins expressed on dendritic cells. The potential for lectin-based antiviral therapies is also discussed.

## 1. Introduction

Lectins are a diverse group of proteins broadly defined as non-immunoglobulin proteins that exhibit high avidity for glycoprotein- and/or glycolipid-associated carbohydrates, but display no enzymatic activity [1]. Genes encoding lectins been identified in all forms of life, including plants, animals, and viruses, an indication of their evolutionary conservation [1]. Indeed, phylogenetic studies indicated that the primitive immune system depended on lectin-protease-mediated opsonophagocytosis [2]. Lectins differ in tissue expression, ligand affinities, structure and function, and are classified by the phylogeny and primary and tertiary amino acid structures of their carbohydrate-recognition domains (CRD). However there are several inconsistencies in this classification system (reviewed in [3]).

Glycosylation is a form of post-translational modification in which glycans—monosaccharides or oligosaccharides—are glycosidically bonded to an organic molecule (reviewed in [4]). This modification plays an essential role in the expression and function of many proteins in eukaryotes and prokaryote cells, with roles in, for example, inter-compound interaction and pathogenic immune evasion. Eukaryotic and viral glycosylation occurs in the host cell endoplasmic reticulum (ER) and Golgi apparatus. Lectin-glycan interactions are generally achieved by hydrogen bonding and Van der Waals forces, and often depend on cations and multivalent interactions between multiple CRDs and multiple, clustered target glycans in order to achieve sufficient affinities for lectin activity [3].

The roles of human lectins include protein modulation, cell growth and homeostasis [4]. As glycoproteins are found on the surfaces of several pathogens to a diverse and widespread degree, some lectins act as pattern-recognition receptors (PRRs), recognising pathogen-associated molecular patterns (PAMPs)—including glycans and nucleic acid—related to invading microorganisms and malignant, apoptotic or dead host cells. This can lead to the induction of an immune response against the invading pathogen. However, the relationship between lectins and viruses is complex. In addition to immune evasion, glycosylation is essential for protein expression, assembly and entry steps in virus replication cycles [5], and many viruses have evolved mechanisms to exploit lectins to enhance infection.

This review focuses on human lectins and their roles during viral infections, concentrating on the well-described lectins mannose-binding lectin (MBL), ficolins and dendritic cell-specific ICAM-3 grabbing non-integrin (DC-SIGN). It highlights the different effects of lectins on viral infections and the consequences of genetic variation in lectin genes on susceptibility to virus infections.

## 2. The Complement Cascade

Complement contributes towards the initial defence against viral infections through a sequential protein activation cascade (reviewed in [6]). There are three known complement activation pathways. Each pathway converges at the formation of C3 convertase, which activates downstream complement factors to constitute the membrane attack complex (MAC). The MAC subsequently forms pores in the lipid membranes of pathogens and infected cells, causing osmotic lysis. Parallel to the cascade, some complement cleavage products mediate inflammation and opsonise pathogens, attracting phagocytes, encouraging antigen aggregation and preventing viral entry. To avoid potentially damaging, excessive complement activity, stringent regulation mechanisms have evolved, including complement factor cleavage and endocytotic shedding of MACs.

**Figure 1:**
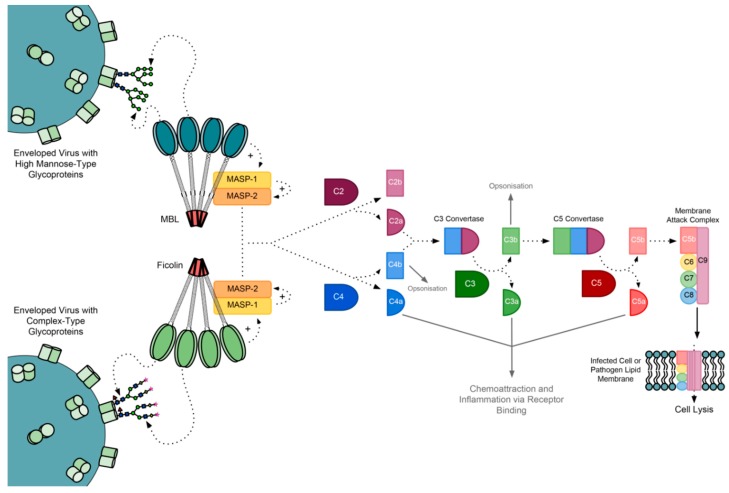
The lectin pathway of complement activation. MBL and ficolins undergo conformational changes upon interaction with viral glycoproteins via glycan-associated mannose and *N*-acetylglucosamine residues, respectively. Sugars are labelled in accordance with reference [7]. This activates MASP-1 followed by MASP-2, which initiates a cleavage cascade of complement factors, with roles in opsonisation, inflammation and pathogen and infected cell lysis.

The alternate and classical complement pathways are triggered by foreign surface molecules and antigen:antibody complexes, respectively. The lectin pathway involves the binding of microbial surface carbohydrate moieties to serum lectins, which activates lectin-bound MBL-associated serine proteases (MASPs) and proteins (MAPs) (Figure 1).

### The MBL-Associated Serine Proteases

MASP-1, MASP-3 and MAP-1 are alternative splice products of the *MASP1* gene [8,9], while MASP-2 and sMAP are encoded by the *MASP2* gene [10]. MASP-1 and MASP-2 cleave C3 and C4, respectively, while C2 is cleaved by both [11]. MASP-2 can produce the C3 convertase C4bC2a and thus activate complement [6] (Figure 1).

Upon ligand binding, lectins undergo a conformational change that brings MASP serine protease domains within close proximity of each other, thus allowing proteolytic autoactivation [12,13,14,15] via the cleavage of an arginine-isoleucine bond in the serine protease domain [16].

MASP-2 activation appears to be primarily dependent on MASP-1, which may autoactivate then *trans*-activate the MASP-2 proenzyme [17]. This might be mediated either by heterodimeric MASP complexes, MBL-MASP-1-MASP-2 co-complexes, or separate MBL-MASP complexes [18,19]. If these complexes are separate, the binding sites of MASP-2 and MASP-1 and -3 on the lectin are likely to be within close proximity and overlapping, but not identical [20].

sMAP and MAP-1 are truncated MASP proteins lacking serine protease domains, and have putative roles in the modulation of MASP-associated complement activation [9,10,21]. MAP-1 may inhibit MASP-2 activation by the disruption of inter-MASP and lectin-MASP co-complexes [18,22].

The MASPs may also associate the lectin pathway of complement with the coagulation system [23,24]. MASP-3 has a putative regulatory function of MASP-2 activity [8], and—due to its involvement in the facial dysmorphic 3MC syndrome—roles for the protein in embryonic development have been hypothesised [25].

## 3. Mannose-Binding Lectin (MBL)

### 3.1. Genetics, Structure, Expression and Binding Specificities of MBL

MBL is a soluble, Ca^2+^-dependent protein of the collectin family–characterised as C-type lectins with collagenous domains–encoded by the *MBL2* gene on human chromosome 10q11.2–10q21 [26]. MBL is primarily expressed in the liver and secreted into the blood, however lower expression has been detected elsewhere, including in mammalian muscle tissue and brain, often following immune challenge [27].

**Figure 2:**
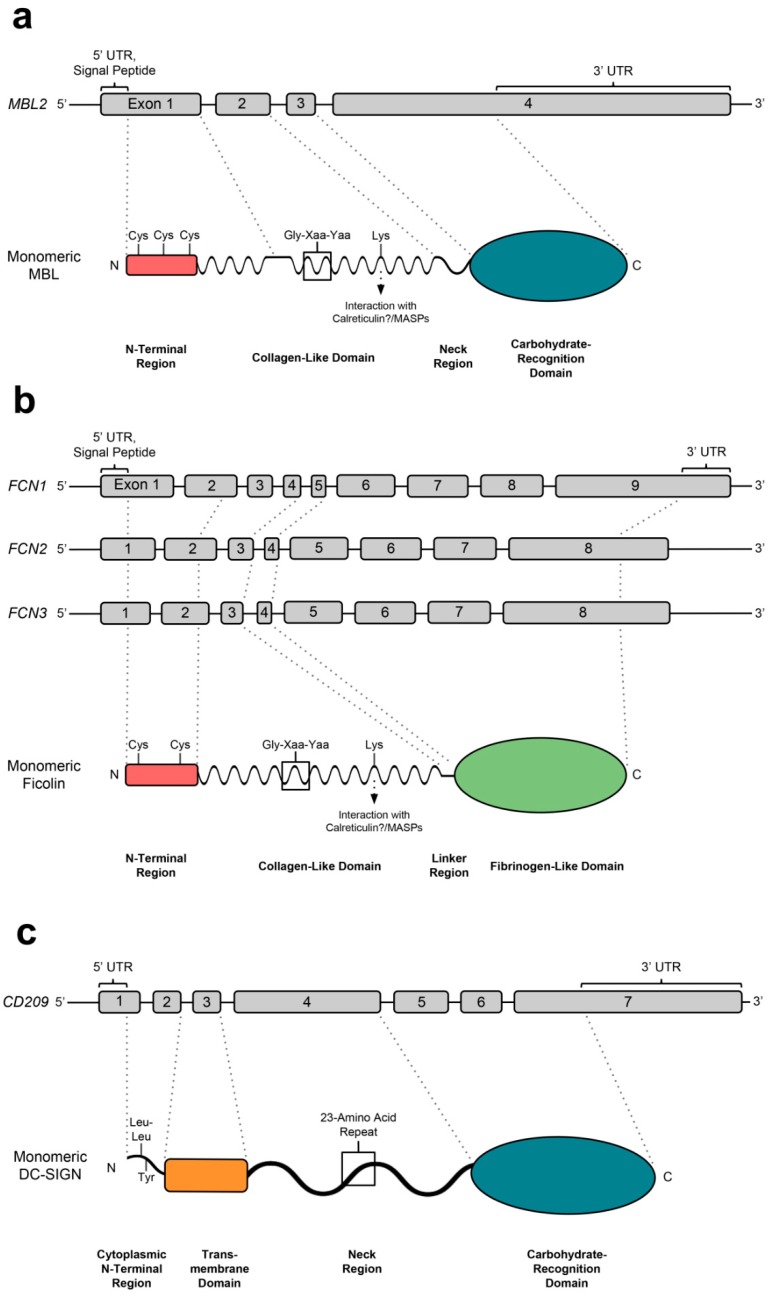
Exon and monomeric structures of (**a**) MBL; (**b**) ficolins; (**c**) DC-SIGN. MBL and ficolins possess 3/2 N-terminal cysteines and a lysine in its CLD, important in oligomerisation and MASP/phagocyte interaction respectively. DC-SIGN contains N-terminal, cytoplasmic dileucine and tyrosine internalisation motifs.

**Figure 3:**
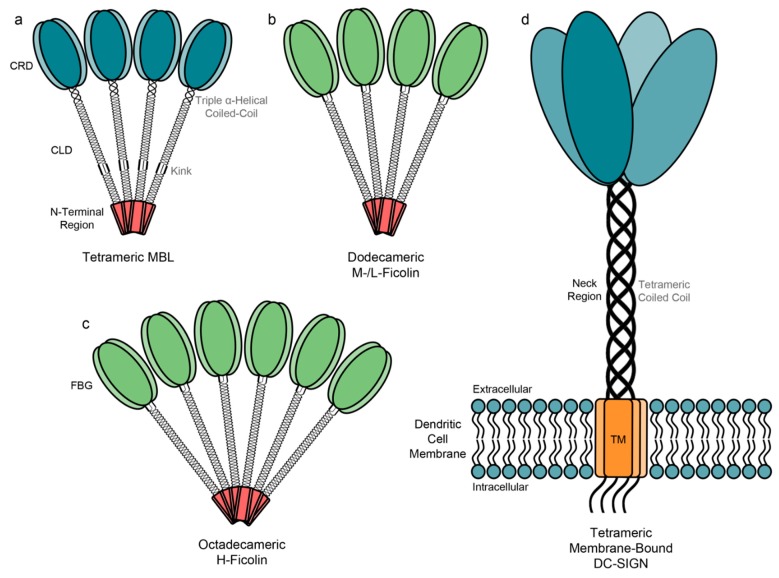
Active, oligomeric structures of (**a**) MBL; (**b**) M-/L-ficolin; (**c**) H-ficolin; (**d**) DC-SIGN. MBL and DC-SIGN possess C-terminal carbohydrate-recognition domains (CRD) whereas ficolins possess fibrinogen-like domains (FBG). MBL and ficolin possess collagen-like domains and consist of trimeric subunits. DC-SIGN possesses a neck region and transmembrane (TM) domain.

MBL monomers are 32 kDa in molecular weight and possess a typical collectin structure: an N-terminal cysteine-rich domain, a collagen-like domain (CLD) of approximately 20 Gly-Xaa-Yaa tandem repeats, a neck region and a CRD responsible for ligand binding (Figure 2a) [28]. These monomers form homotrimeric subunits that further oligomerise into trimeric to hexameric structures that can activate the complement cascade [29]. Trimeric and tetrameric MBL are the most common physiological configurations (Figure 3a) [30].

The CLDs form MBL trimers by hydrophobic interactions, as initiated by α-helical triple coiled coil formation of the neck domain and stabilised by inter- and intra-monomer bridges via 3 N-terminal cysteines [28,30,31]. Oligomeric MBL arranges into a sertiform structure, with trimeric subunits stretching out from the short, bundled N-terminal regions [30,32]. A short amino acid sequence after the first 7 N-terminal Gly-Xaa-Yaa repeats creates a “kink” bend in the CLD, however this is only seen in a minority of MBL molecules *in vivo* and does not influence MBL activity [30,32]. Significant flexibility exists at the CLD-neck and CLD-N-terminus regions, influencing CRD positioning, ligand specificity and MASP interaction [30,32].

MASP binding is centred around a conserved lysine occupying the Xaa position of a CLD repeat [33]. Ligand binding induces a “stretching” event which splays the MBL trimeric subunits and brings MASPs together to enable proteolytic autoactivation [12,13,14,15]. Trimeric/tetrameric MBL may represent the optimal configuration to accommodate MASP auto-activation [30,32].

MBL exhibits specificity for pairs of adjacent equatorial monosaccharide 3- and 4-hydroxyl groups, present in terminal mannose, *N*-acetylglucosamine (GlcNAc), *N*-acetylmannosamine and L-fucose oligosaccharides [34]. This is consistent with the presence of a conserved glutamate-proline-asparagine (EPN) motif in the CRD. MBL also binds phospholipids and nucleic acids, supporting a role in clearance of necrotic tissue [35,36]. MBL may employ higher order oligomers in order to achieve sufficient ligand binding via multivalent bonding [37].

### 3.2. MBL Interaction with Viruses

**Figure 4:**
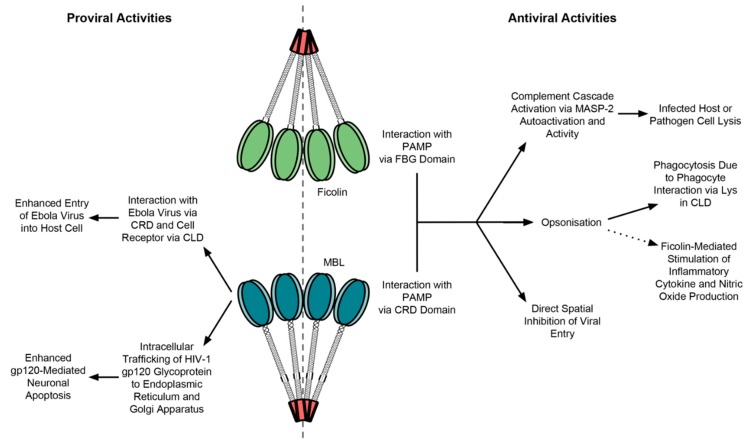
The pro-viral and antiviral activities of serum MBL and ficolins. Both have antiviral activities through complement activation, opsonophagocytosis and direct spatial blocking of virus-receptor interactions and entry. Ficolins also enhance production of inflammatory cytokines and nitric oxide. MBL has specific pro-viral activities in Ebola virus and HIV-1 infections, through enhanced virus entry and neuronal apoptosis respectively.

MBL interacts with several, but not all, viruses in a Ca^2+^-dependent manner. It interacts with several strains of human immunodeficiency virus-1 (HIV-1) via its spatially conserved, high mannose-type, *N*-linked glycosylated gp120 envelope glycoprotein [38]. While HIV-1 uses several mechanisms to evade adaptive immune responses—such as “glycan shielding” in which gp120 glycosylation site mutations prevent neutralising antibody binding but maintain cell receptor binding [39]—MBL is able to bind and neutralise diverse strains of HIV-1. However, MBL does not neutralise HIV-1 through complement activation, even at concentrations far exceeding physiological serum levels [40,41]. Therefore, MBL directly neutralises infection in complement-independent manners, such as opsonisation to enhance phagocytosis by DCs and macrophages, as observed for bacterial infections (Figure 4) [42]. The phagocyte cell surface receptor for MBL has not yet been identified, but a likely candidate is calreticulin—a protein-folding chaperone typically situated in the ER, with roles in antigen presentation—which may bind the MBL at the MASP-binding site [43].

MBL-gp120 interaction also prevents HIV-1 interaction with cell entry inhibitors and inhibits *trans*-infection by direct spatial blocking [40,44]. The same viral glycoprotein-mediated complement-dependent/independent mechanisms are employed by MBL to neutralise influenza A virus (IAV) [45,46], hepatitis C virus (HCV) [47], severe acute respiratory syndrome coronavirus (SARS-CoV) [48,49], Dengue virus (DV) and West Nile virus (WNV) [50,51] infection *in vitro*. Furthermore, MBL can indirectly activate coagulation upon pathogen binding, perhaps via MASP-1 recruitment, as observed in HCV [52] and IAV [45] infection.

#### Viral Exploitation of MBL

Recombinant MBL has therapeutic potential against Ebola virus (EBOV) infection as it neutralises the virus *in vitro* and *in vivo* via complement activation [53], phagocytosis, and direct inhibition of glycoprotein interaction with the DC-SIGN/L-SIGN receptor [54]. However, MBL may have pro-viral effects, enhancing EBOV infection by mediating macropinocytosis in low complement conditions, perhaps via the C1QBP cell receptor (Figure 4) [55]. This may partially explain the high prevalence of allelic variants conferring low MBL levels, as in the context of EBOV lower MBL levels may prevent excessive infection [55]. MBL can also enhance HIV-1 infection of the brain. AIDS-associated dementia complex is characterised by neuronal cell death and cognitive deficits, however HIV-1 infects relatively few brain cells [56]. Instead, HIV-1 sheds gp120 glycoprotein, which is internalised via the CXCR4 receptor on neuronal cells, then bound and shuttled by intracellular MBL in vesicle complexes to the ER and Golgi apparatus, perhaps facilitating gp120-mediated apoptosis (Figure 4) [56,57].

### 3.3. MBL Variants

In addition to the wild-type MBL allele A, 3 MBL variants with single nucleotide polymorphisms (SNPs) in exon 1 are common. These—termed MBL-B, -C and -D—are products of amino acid substitutions in codons 54 [58], 57 [59] and 52 [60] which disrupt Gly-Xaa-Yaa repeats. These cause less stable CLDs, hindering the capacity for trimerisation, MASP-binding and complement activation [20]. The mutations are N-terminal to the CLD kink whereas the MASP-binding sites are C-terminal, indicating that the decreased affinity for MASPs is an indirect result of the destabilisation of the CLD [20].

Certain African and South American populations have low serum MBL levels as a result of high allele MBL-C and -B frequencies, respectively [61]. Frequency of the MBL-D allele is particularly high in African and Caucasian populations [60]. Nevertheless, approximately 10%–30% of the global population are MBL deficient [62]. Heterozygotes for the variant alleles exhibit characteristics of both wild-type and variant homozygotes, including different patterns of oligomerisation and MBL serum levels [63].

However, despite its polymorphisms, the *MBL2* gene and its primate orthologues are highly homologous and conserved, emphasising the evolutionary and immune importance of the protein [64]. Several theories for the high frequencies of various MBL variants with significantly different levels and activities have been proposed, including founder effect, or selective advantages such as heterozygous advantage, decreased complement-mediated tissue damage and a role of MBL in the enhancement of infection [65].

*MBL2* promoter SNPs −550G>C (*H*/*L*), −221C>G (*X*/*Y*) and +4C>T (*P*/*Q*) influence MBL serum concentration [61,66,67]. As a result of linkage disequilibrium, 7 common haplotypes have been described, each with differing serum levels of MBL ranging from <0.01 μg/mL to >5 μg/mL, with an average of 1.7 μg/mL [61,68]. Although alternative immune mechanisms often compensate for MBL defects [69], both excessively high and low serum levels are associated with increased susceptibility to both infectious and autoimmune disease [61]. 

#### The Effect of MBL Variant Alleles on Viral Infection

MBL deficiency-associated alleles correlate with increased susceptibility to infectious diseases, however these relationships are complex (Table 1). Homozygotes and heterozygotes of the variant alleles increase risk of HIV-1 infection [70,71] and disease progression [72]. HIV-1 patients carrying the MBL-B allele had higher viral loads in their sera, likely as a result of decreased MBL-mediated viral elimination [73]. The -221 SNP has been correlated with increased risk of perinatal HIV-1 infection [74], whereas higher MBL levels conferred protection. Low serum level haplotypes of the −550 and −221 SNPs were associated with low CD4^+^ T-cell counts, higher viral loads [75] and accelerated disease progression [72]. In contrast, some studies found no association between variant alleles and susceptibility to HIV-1 infection [76] and disease progression [77,78]. Although MBL levels do not change throughout HIV-1 disease progression, MBL serum levels are elevated in HIV-1-infected patients and are correlated with good response to highly active antiretroviral therapy (HAART) [41,79]. Some studies suggest a protective effect against HIV-1 disease progression conferred by the variant alleles [80].

**Table 1:** Single nucleotide polymorphisms of the *MBL2*, *FCN* and *CD209* genes with associations with virus infections. del = deletion; ins = insertion. +1 represents the A of the ATG translation start site for all genes except MBL2, where +1 represents the transcription start site, to comply with literature.

Gene	dbSNP (Alternative Name)	Nucleotide Position	Major Allele	Minor Allele	Region	Amino Acid Mutation	Relevance to Specific Virus Infections
***MBL2***	rs11003125 (*H/L*)	−550	G	C	Promoter	-	HIV [74,75]
	rs7096206 (*X/Y*)	−221	C	G	Promoter	-	HBV [81], HCV [82,83], HIV [72,75], HTLV [84], SARS-CoV [48]
	rs7095891 (*P/Q*)	+4	C	T	5’ UTR	-	
	rs5030737 (MBL-D)	+223	C	T	Exon 1	Arg52Cys	CMV [85,86], DV[50], HBV [87,88], HCV [82,83,89,90], HIV [70,72,80],
	rs1800450 (MBL-B)	+230	G	A	Exon 1	Gly54Asp	CMV [85,86], DV [50], HBV [81,88,91], HCV [82,83,89,90], HIV [70,72,73,80], HTLV [92], SARS-CoV [48]
	rs1800451 (MBL-C)	+239	G	A	Exon 1	Gly57Glu	CMV [85,86], DV [50], HBV [88], HCV [82,83,89,90], HIV [70,72,80]
***FCN1***	rs2989727	−1981	G	A	Promoter	-	
	rs10120023	−542	G	A	Promoter	-	
	rs28909976	−271	-	InsT	Promoter	-	
	rs10117466	−144	C	A	Promoter	-	Increased serum concentration [93]
	rs10441778	+1435	G	A	Exon 2	Gly43Asp	Likely affects structure and oligomerisation [94]
	ss76901539	+3458	G	A	Exon 4	Arg93Gln	Likely affects structure and oligomerisation [94]
	rs148649884	+6658	G	A	Exon 8	Ala218Thr	Reduced serum concentration, reduced ligand binding [93]
	rs150625869	+7895	T	C	Exon 9	Ser268Pro	Abolished serum concentration [93]
	rs1071583	+7918	G	A	Exon 9	-	
	ss76901546	+7929	G	A	Exon 9	Trp279STOP	Likely affects structure and oligomerisation [94]
	rs138055828	+7959	A	G	Exon 9	Ala289Ser	Reduced serum concentration, reduced ligand binding [93]
	ss76901547	+8000	G	A	Exon 9	Gly303Ser	Likely affects function [94]
***FCN2***	rs3124952	−986	G	A	Promoter	-	Reduced serum concentration [94]; HBV [95]
	rs3124953	−602	G	A	Promoter	-	Increased serum concentration [94]; HBV [95]
	rs17514136	−4	A	G	Promoter	-	Increased serum concentration [94]; HBV [95]
	ss76901565	+4423	C	T	Exon 5	Arg103Cys	Likely affects chemical and structural properties [94]
	ss76901566	+4526	C	T	Exon 5	Thr137Met	Likely affects chemical and structural properties [94]
	ss76901570	+4957	G	A	Exon 6	Arg147Gln	Likely affects ligand binding [94]
	ss76901571	+4987	G	A	Exon 6	Arg157Gln	Likely affects ligand binding [94]
	rs17549193	+6359	C	T	Exon 8	Thr236Met	Reduced binding to GlcNAc [94] and PTX3 [96];
	rs7851696	+6424	G	T	Exon 8	Ala258Ser	Increased binding to GlcNAc [94]; CMV [86], HBV [95]
	rs28357091	+6443_44	CT	A	Exon 8	Ala264fs	Truncated protein [94]
***FCN3***	rs28357092	+1637	C	delC	Exon 5	Leu117fs	Truncated protein [94]; Severe, recurrent respiratory and gastrointestinal infections [97,98,99]
	ss76901551	+1663	A	G	Exon 5	Thr125Ala	Likely affects function [94]
	ss76901555	+5543	T	C	Exon 8	Val287Ala	Likely affects function [94]
***CD209***	rs4804803	−336	A	G	Promoter	-	DV [100,101,102], HCV [103], HIV [104,105,106], SARS-CoV [107]
	rs11465366	−201	C	A	Promoter	-	HIV [104,106]
	rs2287886	−139	T	C	Promoter	-	HIV [106,108]
	rs41374747	+660	G	A	Exon 4	Arg198Gln	HIV [104]
	rs11465380	+791	C	G	Exon 4	Leu242Val	HIV [104]

The MBL variants may also influence Dengue virus infection and disease progression [50]. MBL deficiency is also linked to cytomegalovirus (CMV) reactivation after lung or liver transplantation [85,86] and susceptibility to SARS-CoV [48], yet displayed no correlation with IAV H1N1 infection [109]. Correlations between MBL-B homozygotes and the −221 SNP with susceptibility to human T-cell lymphotropic virus (HTLV) infection have been observed [84,92].

Some studies suggested that MBL variants do not influence susceptibility to HCV infection [110,111] or disease progression [112], however others show an association with HCV infection, disease progression and treatment response [82,83,89,112]. One study associated MBL-B and -D with protection against HCV infection, while associating MBL-C with increased susceptibility [90]. MBL-B is associated with chronic hepatitis B disease progression [91], whereas MBL-D displayed a Caucasian-specific association with hepatitis B virus (HBV) persistence [87]. All variants and the −221 SNP may influence HBV-associated hepatitis, liver cirrhosis and hepatocellular carcinoma [81,88], and MBL serum levels have a role in perinatal HBV infection [113]. However, one study found no correlation between variants and chronic HBV infection [114].

When studying the association of polymorphisms with disease severity, the geographical population, ethnicity, disease severity, asymptomatic patients, age, route of transmission, study techniques and sample size must be taken into account. These confounding factors can, in part, explain discrepancies between studies.

## 4. Ficolins

### 4.1. Genetics, Structure, Expression and Binding Specificities of Ficolins 

Three human ficolins have been described: L-ficolin [115], M-ficolin [116,117] and H-ficolin [118]. Several orthologues have been identified in genetically diverse lineages of animals, an indication of the ancient ancestral origins of these lectins. These include the invertebrate ascidians [119], and vertebrates such chickens [120], non-human primates [121] and pigs [116,122]. The human ficolins differ in several ways, for example in their localisation in the human body and their capacity to trigger an immune response.

The M-, L- and H-ficolin proteins are encoded by the *FCN1*, *FCN2* and *FCN3* genes, respectively, encoding polypeptides of 326, 313 and 299 amino acids, including the signal peptide (Figure 2b) (reviewed in [123]). The *FCN1* and *FCN2* genes are both situated on chromosome 9q34 whereas *FCN3* is found on chromosome 1p36.11. The *FCN2* and *FCN3* genes consist of eight exons, whereas *FCN1* comprises nine exons. 

Each ficolin monomer comprises an N-terminal region with two functionally important cysteine residues, a CLD containing Gly-Xaa-Yaa repeats, a linker region and, characteristically, a C-terminal globular fibrinogen-like domain [124,125]. Like CRDs, the fibrinogen-like (FBG) domain recognises specific pathogen-associated carbohydrates, and the CLD is responsible for signalling to induce an immune response via MASP proteins [124,125]. Active, oligomeric L-ficolin and M-ficolin are dodecamers comprised of four homotrimeric subunits to form what has been labelled as a “bouquet” structure (Figure 3b), whereas H-ficolin is octadecameric (Figure 3c) [124,125,126]. Like MBL, ficolin homotrimers are stabilised by interactions between hydrophobic residues in the CLDs [31,127], and oligomerise by inter-monomer and -trimer disulphide bridges between the N-terminal cysteine residues [124,128].

Hepatocytes are the main site of expression and secretion of both L- and H- ficolin [125,129], although H-ficolin is also highly expressed in type II alveolar and bronchial epithelial cells [129]. Despite minor lung and blood expression, most M-ficolin is associated with the surface of peripheral blood leukocytes [117,130]. H-ficolin is the most abundant serum ficolin (median concentration of ~26 µg/mL; range 6–83 µg/mL) [131] followed by L-ficolin (median of 3.7–5.4 μg/mL; range ~1–13 µg/mL) [132,133] and M-ficolin (median of 1.07 µg/mL; range 0.28–4.05 µg/mL) [134].

All ficolins bind GlcNAc and *N*-acetylgalactosamine (GalNAc) [118,130,135]. H-ficolin also binds GalNAc and D-fucose, but not mannose and lactose [118,135]. M-ficolin also binds sialic acid [130]. These differing ligand specificities are conferred by sequence differences in the binding site—S1—near the Ca^2+^-binding site of the FBG [135]. In addition to S1, L-ficolin has 3 inner binding sites, S2–S4, which exhibit great structural plasticity, thus allowing the sites to accommodate a wide variety of ligands in both Ca^2+^-dependent and -independent ways such as phosphocholine moieties of bacterial teichoic acids, in addition to acetylated compounds [135,136]. For example, S2 is the major binding site for galactose and *N*-acetylcysteine, whereas S3 and S4 cooperate to bind (1,3)-β-d-glucan, among others [135]. 

### 4.2. The Roles of Ficolins in the Immune Response

Like MBL, ficolins indirectly activate the lytic complement pathway via MASP activation, induce phagocytosis by opsonisation, and stimulate the production and secretion of inflammatory cytokines and nitric oxide by macrophages [137]. Interaction with phagocytes is believed to be mediated by a functionally significant lysine in the CLD, at residues 57 and 47 for L- and H-ficolin respectively, which binds calreticulin on phagocyte cell surfaces [126]. The same residue is responsible for interaction with MASPs, therefore it is possible that the phagocytic and complement effects of L-ficolin are competitive [20,126]. L-ficolin may also clear apoptotic and necrotic host cells through the binding of apoptosis-associated ligands [138]. Furthermore, L-ficolin directly prevents viral entry into host cells [139].

### 4.3. Ficolin Interaction with Viruses

The role of ficolins in the clearance of several pathogens has become increasingly evident; however their role in viral clearance requires greater investigation. L-ficolin interacts with viruses via *N*-linked glycans on viral envelope glycoproteins [140,141,142]. L-ficolin binding of HCV triggers infected-cell lysis via C4 deposition, however L-ficolin interaction is abrogated if the HCV E2 glycoprotein is not glycosylated [141]. Biologically relevant levels of recombinant oligomeric L-ficolin, which displayed similar binding activity and structure to serum L-ficolin, neutralised HCV entry in a dose-dependent manner by preventing E2 interaction with cell surface lipoprotein receptor and scavenger receptor B1, which are important for HCV entry [139,143]. Monomeric L-ficolin can activate complement [141] but not inhibit HCV entry [139]. 

Human L-ficolin and porcine ficolin-α neutralise replication and infection of IAV *in vivo* [142] and porcine reproductive and respiratory syndrome virus *in vitro* [140], respectively. L-ficolin directly inhibits IAV entry and promotes complement-mediated lysis of IAV and infected cells [142]. IAV binds sialylated glycans on serum H-ficolin in the airway before viral entry, enabling H-ficolin mediated inhibition of IAV infectivity by direct blocking, viral aggregation and complement activation [144]. As yet unpublished research by Ren *et al*. implicates L-ficolin mediated complement activation following interaction with HIV-1 gp120 [145].

Few M-ficolin interactions with pathogens have been observed, despite its ability to activate complement [130]. M-ficolin inhibits IAV infection [144]. The majority of M-ficolin is monocyte and granulocyte membrane-bound, despite its lack of a transmembrane (TM) domain, and associates with sialylated membranes via its FBG domain [146]. A candidate receptor of M-ficolin is G-protein-coupled receptor 43 (GPCR43) which, upon M-ficolin-mediated pathogenic interaction, indirectly activates IL-8 production [147]. Serum M-ficolin binds sialic acid on capsulated *Streptococcus agalactiae* via its FBG and activates complement, however L- and H-ficolin were found not to interact with this pathogen [148].

### 4.4. Single Nucleotide Polymorphisms in FCN Genes

Hummelshøj *et al*. extensively described the SNPs of the highly polymorphic *FCN* genes (Table 1) [94,149]. In general, polymorphisms in the promoter regions of the ficolin genes are expected to affect gene regulation and protein concentration whereas coding region polymorphisms likely affect protein stability, modification, folding and activity, thus altering protein function. Non-synonymous substitutions alter protein activity, however non-synonymous mutations may influence mRNA processing and protein expression.

While polymorphisms have been identified in the *FCN1* and *FCN3* genes, several more significant SNPs have been identified in the *FCN2* gene. The frequencies of *FCN* polymorphisms often differ between ethnicities, with some existing solely in a particular geographical population, more so in African populations [94,150]. This likely arose from distinct geographical selective pressures, such as genetically-determined and infectious diseases.

The *FCN2* and *FCN3* genes have three and two as yet undetected splicing variants, respectively [115,151]. The *FCN1* gene contains 45 SNPs, nine of which are exclusive to African populations and eight of which are non-synonymous. Gly43Asp, Arg93Gln and Trp279STOP likely affect M-ficolin structure and oligomerisation, whereas Gly303Ser may affect M-ficolin function. The *FCN3* gene showed 15 low frequency SNPs, none of which were found globally. Only Leu117fs, Thr125Ala—corresponding to *FCN2* Thr137Met—and Val287Ala are predicted to affect H-ficolin function. 

Of the 36 SNPs in the *FCN2* gene, five significant SNPs have been identified. Promoter polymorphisms −986A>G, −602G>A and −4A>G affect serum levels of L-ficolin and exon 8 polymorphisms +6359C>T and +6424G>T, conferring Thr236Met and Ala258Ser respectively, in the FBG alter L-ficolin affinity to GlcNAc. Several of these and other polymorphisms were in strong linkage disequilibrium. 

Additional *FCN2* SNPs were detected and their effects hypothesised, however no associated phenotype has yet been described. The Arg147Gln and Arg157Gln mutations are found in the S2 and S3 binding sites respectively, and are therefore expected to affect ligand binding. Furthermore, the Arg103Cys and Thr137Met mutations are expected to affect the chemical and structural properties of L-ficolin. A rare frame shift mutation encoding Ala264fs has also been described, however a homozygote for this polymorphism has not been found, hence the physiological implications are unknown [149]. The rare frame shift SNP +1637delC of *FCN3*, correlating to Leu117fs, encodes a truncated H-ficolin protein that cannot be expressed [97]. This leads to full H-ficolin deficiency—in homozygotes, causing high levels of lung infection and disease, and severe necrotising enterocolitis [97,98,99].

#### 4.4.1. The Significance of *FCN* Gene Single Nucleotide Polymorphisms in Viral Infections

There have been several clinical studies monitoring the part that ficolins play in disease outcome, typically focussing on one or both of two factors: ficolin gene polymorphisms (Table 1) and ficolin serum concentrations. 

One* FCN2* haplotype is associated with protection against HBV infection [95]. L-ficolin levels were higher in patients with acute rather than chronic HBV infection, suggesting that the protein is directly involved in immediate clearance of the virus, and influences subsequent liver disease [95]. The L-ficolin Ala258Ser mutation appears to confer a protective effect against CMV re-infection in liver transplantation when compared to wild-type L-ficolin [86].

The Thr236Met mutation reduced affinity towards pentraxin 3 (PTX3), a serum protein which enhances L-ficolin-mediated complement response to *Aspergillus fumigatus*, suggesting that *FCN2* polymorphisms also alter affinity towards cooperative proteins and thus affect the immune response [96]. Interestingly, the +6424T SNP is associated with low serum levels of L-ficolin, yet also increased L-ficolin binding [133]. It has been hypothesised that this unexpected correlation is due to higher activity and “exhaustion” of the L-ficolin protein as a result of its increased binding affinity. 

Although there are few investigations of the role of L-ficolin in viral infections, *FCN2* polymorphisms were found to be significant in susceptibility to and disease severity of bacterial diseases, including cutaneous leishmaniasis [152], *Mycobacterium* *leprae* [153], *Pseudomonas aeruginosa*-associated bronchiectasis [154], and *Streptococcus pygones*-associated rheumatic fever and chronic rheumatic heart disease [155]. However, the SNPs were not associated with invasive pneumococcal disease [156] and other respiratory tract infections [157]. These studies have been relatively small-scale and geographically limited, and often did not measure the serum concentrations of L-ficolin to confirm the relationship of the polymorphisms and haplotypes with L-ficolin levels, therefore more rigorous larger scale studies would yield more reliable results. 

M-ficolin SNPs have not yet been reported to have roles in viral infections, however the −144C SNP is associated with protection against *M. leprae*-associated leprosy, whereas −1981A, −271delT and −542G correlate with susceptibility, perhaps by altering transcription factor affinity [158]. The −144C SNP has been associated with increased M-ficolin serum levels [93]. The Ala218Thr and Asn289Ser non-synonymous mutations reduced serum levels and ligand binding activity, whereas Ser268Pro abolished serum levels [93]. The −1981A and +7918 SNPs were correlated with rheumatoid arthritis [159]. Interestingly, expression the *FCN1* gene, among others, is up-regulated in chronic HCV patients who possess the CC genotype at the *IL28B* rs12979860 promoter SNP, which is associated with favourable response to pegylated interferon-α and ribavirin treatment [160,161]. This suggests a possible role of M-ficolin in the clearance of HCV, however further studies are needed. No studies have correlated H-ficolin SNPs with infectious diseases.

#### 4.4.2. The Significance of Ficolin Serum Concentrations in Viral Infections 

Serum ficolin levels are dependent on the expressed alleles; homozygotes for particular SNPs exhibit the highest or lowest levels whereas heterozygotes display intermediate levels of ficolin [149]. Specific *FCN2* SNPs that are associated with low levels of L-ficolin tend to cause higher susceptibility to infection [152]. L-ficolin levels are significantly increased in the serum of HCV-infected individuals, and concentrations correlate with the severity of fibrosis [141]. Chronic HCV-associated liver damage also did not reduce the levels of L-ficolin expressed [139]. In chronic HCV-infected patients with abnormal alanine aminotransferase (ALT) levels, serum concentrations of L-ficolin correlated with ALT levels [162]. ALT is a known marker of fibrosis and inflammation [162]. After successful therapy, ALT and HCV RNA levels of these patients all decreased to normal values, followed by a decrease in L-ficolinlevels, suggesting a correlation of ALT and RNA levels with disease outcome, as a result of L-ficolin activity [149,162].

Higher L-ficolin serum concentrations also appear to confer protective effects against microorganism-induced inflammation in allergic respiratory disease [163]. L-ficolin levels were higher in acute severe cases of *Plasmodium falciparum*-based malaria, rather than mild cases [164]. Low H-ficolin serum levels correlate with fever and neutropenia in paediatric cancer patients treated with chemotherapy [131].

### 4.5. Cooperative Relationships between Lectins and Other Immune Proteins

**Figure 5:**
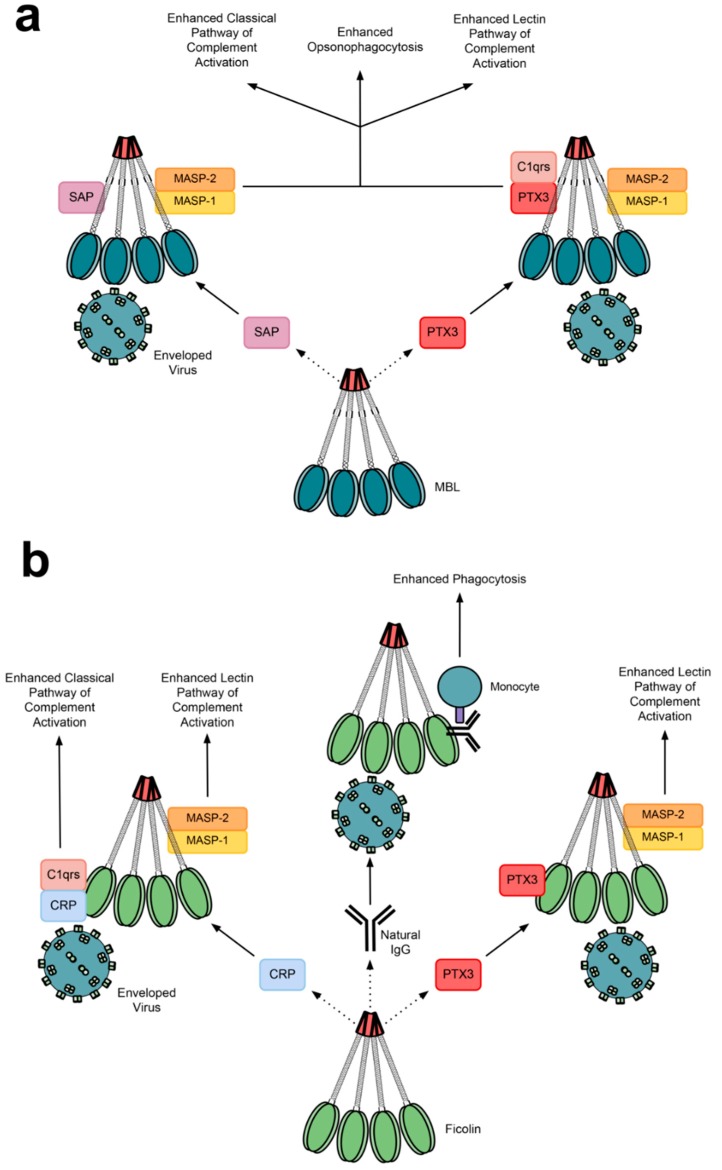
The cooperative antimicrobial relationships of serum (**a**) MBL and (**b**) ficolins with other immune proteins. Complement activation and opsonophagocytosis can be enhanced by SAP and PTX3-C1q interaction with MBL, and PTX3, CRP-C1qrs and natural IgG interaction with ficolins.

A consequence of complement activation is the subsequent activation of the humoral immune system against a pathogen, thus enhancing the adaptive immune response and memory [6]. Lectins can also directly interact with immune components and enhance the antimicrobial response. MBL can bind the serum PRRs PTX3 and serum amyloid P component (SAP) via its CLD to promote complement activation and opsonophagocytosis of *Candida albicans* (Figure 5a) [165]. PTX3 required C1q to enhance activation of the classical complement pathway [165]. 

Natural antibodies are produced without prior exposure to infection or immunisation, and are important in the protection of individuals exposed to pathogens for first time, such as neonates [166]. They are able to initiate complement alone, however under mild acidosis and reduced calcium levels—conditions found at infection-inflammation sites—an additional binding site is exposed on the ficolin FBG to allow complex formation with natural immunoglobulin G (nIgG) [166,167]. This allows indirect nIgG-based phagocytosis via L-ficolin opsonisation, leading to a stronger immune response (Figure 5b) [166]. PTX3 interacts with L-ficolin to enhance its binding of *Aspergillus fumigatus* and its induction of C4 deposition [96]. Similarly, M-ficolin binds sialic acid on PTX3 in a Ca^2+^-dependent manner via its FBG domain [168,169]. M-ficolin:PTX3 complexes enhance phagocytosis of apoptotic and necrotic cells [169]. H-ficolin may also cooperate with PTX3 [144], however specific interaction between the two has not been reported [96,168].

Similar to nIgG:ficolin complexes, infection-inflammation conditions significantly increase interaction between the acute phase protein C-reactive protein (CRP) and L-ficolin, leading to a stronger classical- and lectin-mediated complement response against *Pseudomonas aeruginosa* [170]. A pH- and calcium-sensitive binding site on the ficolin FBG domain enables binding to CRP [171]. Later phase infection-inflammation conditions also enhance interaction between membrane GPCR43-associated M-ficolin and CRP, thus blocking M-ficolin binding of PAMPs and curtailing GPCR43-mediated IL-8 production, and allowing the restoration of homeostasis upon infection and injury [147,171].

## 5. DC-SIGN

### 5.1. Genetics, Structure, Expression and Binding Specificities of DC-SIGN 

Dendritic cells (DCs) express PRRs, such as DC-SIGN [172], which play a role in antigen capture and internalisation. This contributes to maturation and migration of DCs to secondary lymphoid organs where they present antigen to resting T-cells [173]. The DC-T-cell interaction is mediated by the transient interaction of DC-SIGN with ICAM-3 on the T-cell surface [174]. 

Human DC-SIGN—encoded by the CD209 gene—is a C-type lectin, the monomeric structure of which comprises a C-terminal, Ca^2+^-dependent CRD followed by a flexible neck region which forms an α-helical coiled coil upon trimerisation and which consists of 7.5 23-amino acid repeats involved in oligomerization [175,176]. A TM region follows, then a cytoplasmic N-terminal region responsible for internalisation via di-leucine and tyrosine motifs (Figure 2c) [175,177]. Adjacent to *CD209* on chromosome 19p13 is the *CD299* gene, encoding the membrane-bound C-type lectin L-SIGN (DC-SIGNR)—a protein that is highly homologous to DC-SIGN [178]. This protein performs similar roles to DC-SIGN, but differs in tissue distribution and expression, being expressed mainly in liver and lymph nodes.

Active, tetrameric DC-SIGN (Figure 3d) is organised on DC surfaces in nanoclusters, mediated by the neck region and permitting interactions with pathogens of a variety of sizes, as large as 300 nm across as seen with the measles virus (MV) [175,176]. The level of DC-SIGN expression is important to optimal virus binding, which partially explains the relatively more efficient HIV-1 binding exhibited by DCs rather than other DC-SIGN^+^ cells [179,180]. Furthermore, the virus-binding capacity of DC-SIGN is also governed by the cell type in which the virus replicated, as each cell type causes subtle changes in the glycosylation profile of viral glycoproteins [181].

DC-SIGN preferentially binds fucosylated and high mannose-type oligosaccharides using different binding sites, with differing avidity depending on the configuration of the mannose and fucose residues in the glycan ligand, as well as the presentation of the target molecule on the pathogen surface [175].

### 5.2. Exploitation of DC-SIGN by Viruses

**Figure 6:**
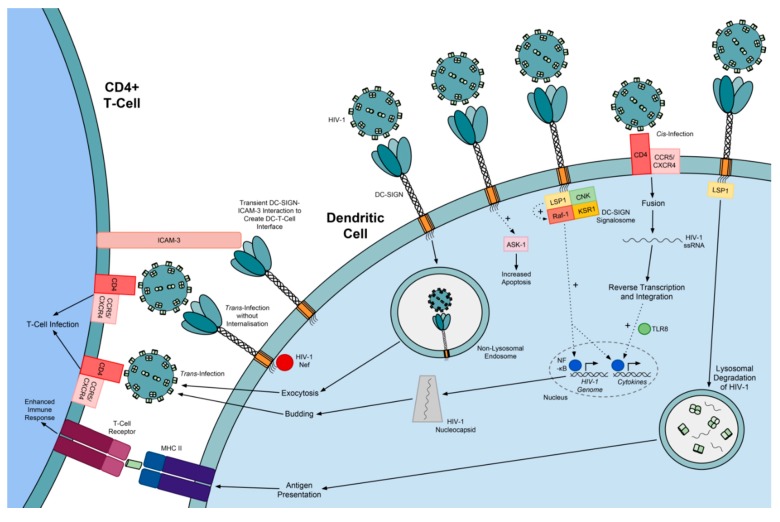
The role of DC-SIGN in HIV-1 infection. DC-SIGN neutralises HIV-1 infection through increased DC-SIGN signalosome-mediated cytokine production and degradation of virions. DC-SIGN then aids dendritic cell-T-cell interaction through transient ICAM-3 binding, thus allowing antigen presentation to T-cells to enhance the immune response. However, HIV-1 exploits DC-SIGN to increase dendritic cell apoptosis via ASK-1, enhance viral replication via the DC-SIGN signalosome and to evade the immune response in specialised non-lysosomal endosomes. DC-SIGN also enhances HIV-1 *trans*-infection of T-cells.

DC-SIGN is exploited by several pathogens for host cell binding and entry. The current, generally accepted model of DC-SIGN-mediated HIV-1 infection utilises DC-SIGN^+^ cells, such as DCs, to transport virions from sites of HIV-1 exposure—at the mucosal membranes or bloodstream—to CD4^+^ T-cell targets in the lymphoid tissues (reviewed in [182]). In addition to the primary HIV-1 receptor CD4 and CCR5 or CXCR4 co-receptors [183], DC-SIGN also recognises HIV-1 gp120 [172], resulting in the activation of downstream processes. Most HIV-1 virions are shuttled to the proteasome, aided by the interaction of the cytoskeletal phosphoprotein LSP1 with HIV-1-bound DC-SIGN, where it is degraded for MHC class II presentation to T-cells [184,185]. Alternatively, HIV-1 exploits the DC-SIGN signalosome—comprising DC-SIGN, LSP1, KSR1 and CNK—to activate Raf-1 and modulate cytokine response, and to enhance NF-κB-mediated transcription of the HIV-1 genome [186]. Independently, HIV-1-bound DC-SIGN modulates TLR-induced cytokine and HIV-1 genome expression (Figure 6) [186]. 

DC-SIGN binding to HIV-1 can also enhance CD4-gp120 glycoprotein interactions and CCR5-mediated entry [187,188]. As a result, DC-SIGN-bound, intact HIV-1 virions are non-fusogenically internalised into a specialised, non-lysosomal, low-pH endosome, where virions are able to maintain stability and infectivity for several days without the need for replication [177,189]. Whether an HIV-1 virion is degraded or maintained in a DC is dependent on several factors, including the *N*-linked glycan composition of gp120 [190].

DC-internalised, stable HIV-1 is can be transferred to T-cells [177]. Upon DC-T-cell contact, protected HIV-1 virions, adhesion molecules and receptors rapidly accumulate at the DC-T-cell interface, enabling HIV-1 transfer across the infectious synapse and gp120 binding to T-cell receptors [191]. HIV-1 Nef may prevent DC-SIGN-bound HIV-1 internalisation to enhance *trans*-infection [192,193]. *Trans*-infection is more likely to occur in more mature DCs, which are more efficient at protecting the virus by endocytosis whereas immature DCs are more efficient at degradation [184,191]. In contrast, HIV-1 binding to DC-SIGN may slow DC maturation by—for example—reducing expression of CD86 and MHC class II, in order to prevent immune response and prime the cell for *trans*-infection [194]. Other lectins are implicated in HIV-1 *trans*-infection, such as the mature DC-expressed sialic acid-binding Ig-like lectin 1 (Siglec-1), which binds sialyllactose-containing gangliosides on the HIV-1 surface [195]. Interestingly, a feature of acquired immunodeficiency syndrome (AIDS) is the gradual depletion of DC levels, increasing the risk of opportunistic infection [196]. This is a result of gp120-DC-SIGN interaction in certain conditions to prime the DC for apoptosis, through excessive activation of the pro-apoptotic kinase ASK-1 [196].

Several other viruses utilise DC-SIGN for different purposes. For example, many viruses use DC-SIGN as an attachment factor and for *trans*-infection, such as MV [197], EBOV [198], SARS-CoV [199], IAV [200], HCV [201] and CMV [202]. Others use DC-SIGN for fusion and internalisation, such as human herpesvirus-8 [203] and HCV [204]. L-SIGN is similarly exploited by many viruses as a glycoprotein-mediated attachment and internalisation receptor, as is observed with HIV-1 [205], HCV [201], SARS-CoV [206], Marburg virus [206] and EBOV [198]. However, DC-SIGN and L-SIGN differ in viral interaction and polymorphisms, as the neck region of L-SIGN is highly variable and polymorphic whereas DC-SIGN is generally conserved [207]. Furthermore, L-SIGN binds and promotes infection of certain viruses more efficiently than DC-SIGN, for example WNV [208].

### 5.3. DC-SIGN Variants 

Several DC-SIGN isoforms exist as a result of alternative splicing, resulting in both membrane-bound and soluble forms with alternative cytoplasmic and CRD regions, missing TM domains and variable neck domain repeat regions [209]. Relatively little is known of the physiological and immune roles of soluble DC-SIGN (sDC-SIGN). Tetrameric sDC-SIGN has been detected in bodily fluids and the cytoplasm of some DC-SIGN^+^ cells, however it is unknown how the protein is secreted [209,210]. Interestingly, CMV interacts with sDC-SIGN via its gB glycoprotein, in the same manner as its interaction with membrane-bound DC-SIGN, possibly to enhance viral uptake by DCs [210].

Considerable purifying selection pressure has been exerted on the *CD209* region encoding the DC-SIGN neck region, preventing the accumulation of polymorphisms in the global population and maintaining the typical neck region size of 7.5 repeats [207]. In fact, the *CD209* gene only displays 2% variant heterozygosity, and the prototypic 7.5 neck region repeat allele is present in 99% of the global population [207]. This length and the tetrameric structure constrain the CRDs and partially influence specificity for certain viruses and glycan structures with certain geometric configurations [175]. Despite this conservation, eight low frequency *CD209* alleles encoding DC-SIGN proteins with two to 10 neck region repeats have been documented [207]. Different DC-SIGN isoforms can be found on the same cell surface and can affect DC-SIGN multimerisation, and therefore could, in theory, influence susceptibility to HIV-1 infection [211].

### 5.4. The Significance of DC-SIGN Variants in Viral Infection

The Leu232Val and Arg198Gln mutations of DC-SIGN enhanced HIV-1 capture and *trans*-infection [104]. While some studies have identified no significant DC-SIGN exon 4 neck region SNPs in the context of HIV-1 [212] it has been observed that DC-SIGN variants with less than five neck region repeats are rare but are more frequent in Chinese populations, and are associated with protection against HIV-1 transmission [213]. Heterozygotes for variants with atypical numbers of neck region repeats above five were also associated with protection against HIV-1 infection [214]. CMV has been observed to interact with sDC-SIGN via its gB glycoprotein, in the same manner as its interaction with membrane-bound DC-SIGN, possibly to enhance viral uptake by DCs [210].

*CD209* promoter SNPs have been implicated in resistance and susceptibility to several infectious diseases, however such correlations are often limited to particular geographical populations. The −336G SNP and AA genotype confer protection against DV-based dengue fever [100,101], perhaps due to reduced transcription factor binding and activity [107], decreased susceptibility of DCs to infection [101] and the suppression of symptoms [102]. The −336 GG/AG genotypes, on the other hand, increase susceptibility to dengue haemorrhagic fever as a result of higher DC-SIGN expression and immune activity against DV [100]. The −139G allele is associated with protection against HIV-1 infection [108], whereas −336C is implicated in higher susceptibility to parenteral HIV-1 infection [105]. The −139, −201 and −336 SNPs are also implicated in perinatal transmission of HIV-1 [104,106]. The −336 SNP influenced liver disease severity in HCV patients [103] and the −336 AG/GG genotypes pre-empted improved prognosis for SARS-CoV patients [107].

## 6. Additional Lectins with Pro-Viral and Antiviral Properties 

In addition to DC-SIGN, several membrane-bound lectins are exploited by viruses to enhance infection, such as HIV-1 exploitation of the C-type lectin mannose receptor [215,216] and Siglec-1 [195], however there are significantly less soluble lectins involved in enhancing infection. Nevertheless, a few candidates, in addition to MBL, have been identified.

The soluble pulmonary collectins SP-A and SP-D both inhibit IAV haemagglutinin activity by viral aggregation and enhancement of the neutrophil immune response [217]. SP-A also has roles in enhancing phagocytosis via the host SP-A receptor 210, which may be exploited by IAV to cause excessive lung inflammation [218]. Furthermore, SP-D can bind and neutralise several strains of HIV-1 through virion agglutination and spatial blocking of gp120-CD4 interaction [219]. However, SP-D enhances HIV-1 *trans*-infection from DCs to T-cells [220]. Similarly, SP-A prevents HIV-1 *cis*-infection of CD4^+^ T-cells, yet enhances *cis*-infection of DCs and *trans*-infection of T-cells [221]. SP-A also enhances entry and fusion of respiratory syncytial virus (RSV) [222].

Galectins are soluble lectins of varying structure and oligomerisation that exhibit specificity for glycans containing β-galactoside [3]. Galectin-1 binds both HIV-1 gp120 glycans—through its CRD—and human cell CD4 receptor, thus allowing the protein to directly stabilise and cross-link HIV-1-CD4 interaction and enhance viral attachment and entry [223]. Similarly, galectin-9 cross-links HIV-1 interaction with cell surface-associated protein disulphide isomerase to enhance fusion and entry [224]. Galectin-3, on the other hand, intracellularly bridges HIV-1 Gag p6 interaction with ALG-2-interacting protein X (Alix) to promote HIV-1 budding [225].

Membrane-associated lectins are generally described to be exploited by viruses for entry. However, some can have significant roles in viral clearance. The C-type lectin langerin is expressed on the surface of Langerhans cells, an epithelial DC subset, and binds the mannose-containing glycans of gp120 [226]. Unlike DC-SIGN, langerin mediates internalisation into Birbeck granules for viral degradation and antigen presentation while efficiently preventing HIV-1 transmission [226]. Langerin is also responsible for the capture, but not internalisation, of MV [227].

## 7. Lectin Therapy for Viral Infections

### 7.1. Soluble Lectin Therapy

Virus-associated glycans are emerging as potential targets for antiviral therapy [7]. Preliminary clinical trials of regular MBL replacement therapy for MBL-deficient patients using plasma-purified MBL have been attempted, and resulted in normal, long-term complement activation and opsonisation activities with no obvious adverse or autoimmune effects [228]. The production and purification of safe, active and functional therapeutic MBL is feasible, but requires optimisation [229]. Therapy using recombinant MBL avoids ethical issues and allows cost-effective large-scale production [230], and phase I trials proved recombinant MBL to be tolerable, safe and effective in the restoration of MBL activity in MBL-deficient patients, with mild to no adverse effects [231].

Due to its complex structure, recombinant active MBL is complicated and expensive to produce [232], whereas recombinant chimaeric lectins (RCLs) can exhibit superior protective potency, cost-effectiveness and safety [217,230,231,233]. The antiviral efficacy of RCLs consisting of the structurally similar lectins MBL and L-ficolin has been studied [232,234]. Like L-ficolin, active MBL/L-ficolin RCLs formed dodecamers, and RCL2 (L-FCN/MBL76) exhibited stronger MBL CRD-mediated ligand binding, likely due to enhanced CRD flexibility [232]. RCL2 possessed a MASP- and calreticulin-binding site consisting of both MBL and L-ficolin fragments, and displayed strong complement activation and opsonophagocytic properties [232]. Physiological levels of RCL2 neutralised EBOV pseudotype infection, and moderately neutralised Hendra and Nipah virus infection [232]. RCL2 and RCL3 (L-FCN/MBL64)—which possesses the MBL MASP-binding site and kink—exhibited anti-IAV activity *in vitro* by enhancing complement activation, inducing viral aggregation, inhibiting IAV envelope glycoprotein activities and, as a therapeutic advantage, exhibiting a reduced association with MASP-1 to diminish coagulation system activities [235]. RCL3 demonstrated more efficient anti-IAV effects *in vivo* by maintaining a cytokine and inflammatory balance that is more advantageous for the host [234].

Similar therapeutic approaches for other lectins have been researched. For example, specific mutagenic engineering of human SP-D enhanced IAV binding and clearance and murine survival *in vivo* [236]. Porcine SP-D neutralises a wider range of IAV infections more potently than recombinant human SP-D *in vitro* and *ex vivo*, however it may be immunogenic in humans, therefore further development is necessary [237]. 

Although ficolins have not been used clinically as antiviral therapies, given the promise of MBL therapy there are is the potential for therapeutic administration of ficolins for virus infections. Due to the essential and conserved nature of *N*-linked glycosylation sites in the life cycles of many enveloped viruses, acetylated sugars are potential targets for direct entry inhibition and viral clearance by L-ficolin administration. Indeed, there are no documented HCV strains resistant to the antiviral effects of the ficolins [238]. Ficolins could be used in combination with antibody therapy. However, the degree to which antibodies interact with these complement components varies. For example, MBL prevents, rather than enhances, the HIV-1 neutralising activity of the 2G12 antibody [239]. Moreover, the importance of complement in the protection against infectious disease varies with each pathogen and each strain. For example, HCV genotypes with more heavily glycosylated E1E2 glycoproteins appear to be more susceptible to lectin-mediated neutralisation [47]. 

Interference of the immune system, in particular the complement system, is extremely complex and may lead to potentially harmful and excessive immune activity, therefore strict caution and testing should be imposed [240]. Passive immunotherapy does not actively trigger an immune response to fight infection, and avoids excessive stimulation of the complement system [139]. Therefore L-ficolin could solely act as an entry inhibitor, after site-directed mutagenesis at the Lys-57 residue of the FBG domain, which is responsible for MASP interaction and interaction with phagocytic receptors [126]. 

### 7.2. Therapy Using Xenogeneic Lectins

An alternative approach is the use of carbohydrate-binding agents (CBA), which interact with viral glycoproteins in order to inhibit DC-SIGN interaction. These tend to be xenogeneic, such as the algal and cyanobacterial lectins griffithsin (GRFT), cyanovirin-N (CV-N), scytovirin (SVN), *Oscillatoria agardhii* agglutinin (OAA) and the synthetic antibiotic pradimicin S, which act as CBA inhibitors of HIV-1 interaction with DC-SIGN *in vitro*, *in vivo* and *ex vivo* [241,242,243,244,245,246]. These lectins therefore have potential uses in antiviral microbicides and have elicited non-toxic and non-immunogenic HIV-1 neutralisation in mammals *in vitro* and *in vivo* [241,244,247]. OAA and the hybrid OPA molecule (a synthetic chimera with the *Pseudomonas fluorescens* agglutinin) are notable as uniquely they interact with oligosaccharide Man-9, rather than terminal mannoses of Man-8/9 [248].

GRFT also has antiviral activity against HCV [249], SARS-CoV and several coronaviruses *in vitro* [250]. *In vivo*, GRFT reduced viral titres and pathology in Japanese encephalitis-infected, HCV-infected and SARS-CoV-infected mice when administered intraperitoneally, subcutaneously and intranasally, respectively [250,251,252]. CV-N inhibited entry by HCV [253], MV and human herpesvirus 6 [254]* in vitro* and prevented further infection and mortality of IAV-infected mice and ferrets when administered intranasally, however it was ineffective when administered subcutaneously [255]. Nevertheless, several CBAs have small viral target ranges, likely as a result of diverse viral glycoproteins. For example, certain CBAs inhibited HIV-1 and HCV infection, yet were ineffective against Herpes simplex virus, vesicular stomatitis virus, RSV, parainfluenza virus-3 [256], adenovirus type 5, CMV, herpesvirus type 1 [257] or vaccinia virus [254]. 

Mitogenic activity, which affects cytokine expression, and a short plasma half-life are characteristic of non-human immune proteins used in the human milieu [243,258]. To overcome this, a linker-extended and PEGylated CV-N was produced in order to extend the protein’s half-life, dampen its immunogenicity and cytotoxicity, and yet maintain its antiviral activity [258]. 

GRFT elicits a far greater anti-HIV-1 effect than CV-N and SVN, and does not cause significant mitogenic, inflammatory, cytotoxic or irritant effects [259,260]. Despite the possibility that GRFT is not sufficiently stable for therapeutic administration [260], subcutaneously-administered GRFT remained active in mice for several days after injection, with minimal adverse effects [259].

## 8. Conclusions

With their ancient origins and multiple physiological functions, lectins are part of the evolutionary arms race between the human immune system and infecting viruses. Lectins have evolved the ability to bind diverse ligands and limit viral infections through a range of immune activities. Viruses have evolved highly glycosylated proteins that are able bind lectins to enhance their attachment, entry and transmission, yet can evade lectin-mediated neutralisation. Despite viral exploitation, lectins have the potential to prime the immune system and enhance viral clearance. This emerging field has already yielded novel antiviral therapies, but our understanding of the specificity of these lectins and the virus-host dynamics remains incomplete. Improved understanding of these interactions will aid our development of these proteins as antiviral therapeutics.

## Abbreviations

CRD: carbohydrate-recognition domain
PRR: pattern-recognition receptor
PAMP: pathogen-associated molecular pattern
MBL: mannose-binding lectin
DC-SIGN: dendritic cell-specific ICAM-3 grabbing non-integrin
MAC: membrane attack complex
MASP: MBL-associated serine protease
MAP: MBL-associated protein
CLD: collagen-like domain
GlcNAc: *N*-acetylglucosamine
SARS-CoV: severe acute respiratory syndrome coronavirus
DV: Dengue virus
WNV: West Nile virus
EBOV: Ebola virus
HAART: highly active antiretroviral therapy
CMV: cytomegalovirus
HTLV: human T-cell lymphotropic virus
FBG: fibrinogen-like
GalNAc: *N*-acetylgalactosamine
GPCR43: G-protein-coupled receptor 43
PTX3: pentraxin 3
SAP: serum amyloid P component
nIgG: natural immunoglobulin G
CRP: C-reactive protein
DC: dendritic cell
MV: measles virus
Siglec-1: sialic acid-binding Ig-like lectin 1
sDC-SIGN: soluble DC-SIGN
RCL: recombinant chimaeric lectin
CBA: carbohydrate-binding agent
GRFT: Griffithsin
CV-N: Cyanovirin
SVN: Scytovirin
OAA: *Oscillatoria agardhii* agglutinin
